# Novel anti-ITGA4 monoclonal antibody induces cell death via large pore formation in NK/T-cell lymphoma cells

**DOI:** 10.1038/s41598-025-32892-0

**Published:** 2025-12-30

**Authors:** Shiori Takeuchi, Yasuhiko Ito, Shuji Matsuoka, Masaaki Abe, Hiromichi Tsurui, Yoshiya Horimoto, Takeshi Fukuhara, Takeshi Hirano, Natsuko Mizutani, Takumi Ito, Ryo Hatano, Atsuhito Nakao, Yasuhisa Terao, Mari Kitade, Yoshitomo Hamano, Hiroyuki Takamatsu, Hiroshi Matsuoka, Tetsuya Nakatsura, Hideo Yagita, Ko Okumura, Atsuo Itakura

**Affiliations:** 1 Department of Obstetrics and Gynecology, Graduate School of Medicine, Juntendo University, Tokyo, Japan; 2 Department of Immunological Diagnosis, Graduate School of Medicine, Juntendo University, 2-1-1 Hongo, Bunkyo-ku, Tokyo, 113-8421 Japan; 3 Division of Cancer Immunotherapy, Exploratory Oncology Research & Clinical Trial Center, National Cancer Center, Kashiwa, Chiba Japan; 4 Department of Breast Surgery and Oncology, Tokyo Medical University, Tokyo, Japan; 5 Neurodegenerative Disorders Collaboration Laboratory, RIKEN Center for Brain Science, Wako, Japan; 6 Department of Neurology, Graduate School of Medicine, Juntendo University, Tokyo, Japan; 7 Department of Medical Technology, Faculty of Health Science, Kyorin University, Tokyo, Japan; 8 Department of Therapy Development and Innovation for Immune Disorders and Cancers, Graduate School of Medicine, Juntendo University, Tokyo, Japan; 9 Department of Immunology, Faculty of Medicine, University of Yamanashi, Yamanashi, Japan; 10 Faculty of Transdisciplinary Sciences for Innovation, Institute of Transdisciplinary Sciences for Innovation, Kanazawa University, Kanazawa, Japan; 11 Division of Medical Oncology/Hematology, Department of Medicine, Kobe University Graduate School of Medicine, Kobe, Japan; 12 Atopy (Allergy) Research Center, Graduate School of Medicine, Juntendo University, Tokyo, Japan

**Keywords:** Lymphoma, Cell death and immune response

## Abstract

Natural killer (NK)/T-cell lymphomas are a highly aggressive lymphoma subtype common in East Asia and Latin America. To develop a therapeutic monoclonal antibody (mAb) against it, BALB/c mice were alternately immunized with two vigorous NK lymphoma cell lines. After hybridization, culture supernatants of the hybridoma clones were added to a third NK lymphoma cell line not used for immunization, and the antibodies were screened for direct cytolytic activity. Results showed that the newly established mAb, named mAb ANAP, induced immediate cell death against NK lymphoma cells in a cytoskeleton-dependent manner, which was also complement-, antibody-dependent cell-mediated cytotoxicity-, and caspase-independent, forming large pores on target cell surface within 20 min; mAb ANAP did not injure normal cells and could bind to the ITGA4 (CD49d). Contrary to existing anti-ITGA4 antibodies, which did not exhibit any destructive activity against NK lymphoma, ANAP antibody has promising potential as a therapeutic agent for NK lymphoma.

**Supplementary Information:**

The online version contains supplementary material available at 10.1038/s41598-025-32892-0.

## Introduction

Novel antibody therapies, including checkpoint inhibitors^1^, cytotoxic antibodies^2^, and chimeric antigen receptor (CAR)-T therapy^3,4^, have revolutionized the treatment of hematologic cancers^5–7^. When it comes to malignant lymphomas, brentuximab, an anti-CD30 monoclonal antibody (mAb), and rituximab and obinutuzumab, which are anti-CD20 antibodies, have been used to treat Hodgkin lymphoma and B-cell lymphomas, respectively^2,8–10^. Previous studies also showed that the addition of rituximab to rituximab plus cyclophosphamide, doxorubicin, vincristine, and prednisone (CHOP) chemotherapy (RCHOP) can significantly improve disease-free survival and overall survival compared to CHOP alone as a first-line therapy for patients with B-cell non-Hodgkin’s lymphoma and lymphocytic leukemia^6,8,10^. Mogamulizumab, an anti-CC chemokine receptor 4 antibody, is effective against adult T-cell leukemia/ lymphoma (ATL), and mogamulizumab monotherapy exhibits clinically significant effects in patients with relapsed aggressive ATL, while at the same time it maintains an acceptable toxicity profile^11,12^. Additionally, newer agents, such as immunotoxin-linked antibodies, radio-labeled antibodies, and antibodies against novel target antigens, are showing promising potential in phase I and II trials across various clinical settings for treating different types of lymphoma and leukemia^13^.

However, despite their aggressive nature, there is currently no successfully mAb therapy available for natural killer (NK) or NK/T-cell lymphomas and leukemias^14^. Although NK lymphoma and leukemias show a peculiar geographic predilection for Asian and South American populations, they are extremely rare diseases^15^. Among them, the extranodal NK/T-cell lymphoma (ENKL) nasal type is the most lethal and invasive neoplasm^16^. In Japan, ENKL has been historically referred to as Rotten nose disease, owing to its rapid invasion and destructive lesions caused in the nasal cavity and surrounding structures that could destroy the center of the face. As a result, necrosis is usually extensive, dissemination to various sites is rapid, and prognosis is very poor^17^. This type of lymphoma cell is resistant to anthracycline-based standard chemotherapy, and apoptosis is only induced by L-asparaginase^18^. More specifically, the L-asparaginase-containing regimen dexamethasone, methotrexate, ifosfamide, L-asparaginase, etoposide (SMILE) has been shown to prolong survival in advanced ENKL^19,20^. However, even with SMILE therapy, the 5-year overall survival remains under 50%^21^. Another factor in ENKL prognosis is the programmed cell death pathway^22^. Relapsed/refractory NK/T-cell lymphoma show rather higher complete remission rates after blockade with pembrolizumab (anti-PD-1 mAb)^23^. Since no other effective salvage treatments are currently available, the development of a novel therapy for NK lymphoma patients is an urgent priority.

Most of the effective mAbs that are currently used in clinical settings bind to cancer or immune cells. Most therapeutic mAbs against cell surface molecules exert their effects mainly through classic immunological mechanisms, including complement-dependent cytotoxicity (CDC), antibody-dependent cellular cytotoxicity (ADCC), antibody-dependent cell-mediated phagocytosis (ADCP), and induction of apoptosis by engagement of specific cell ligands^24^. In lymphoma/leukemia patients, therapeutic mAbs may be less effective due to immunosuppression caused by radiation therapy, chemotherapy, or the malignancy itself, thereby reducing the efficacy of mechanisms such as ADCC or ADCP. However, some therapeutic mAbs can directly induce programmed cell death, and certain reports have shown that therapeutic antibodies with Fc-independent cytotoxic activity can induce non-apoptotic cell death^25–28^. Additionally, some mAbs are noted for their ability to induce membrane lesions^25,28–31^.

Previously, we developed several cytolytic mAbs, including RE2, an anti-mouse pan MHC class I mAb^29,30^, and mAb 4713, an anti-pan HLA class II mAb^28^. These antibodies selectively induce direct cell death in lymphoma cells, while at the same time sparing normal resting lymphocytes.

In this study, we developed a novel mAb, named ANAP, which induces direct cell death in NK and NK/T lymphomas. We selected hybridoma clones based on the direct cytolytic activity of their supernatants against NK lymphoma cells, intentionally excluding ADCC, ADCP, and CDC-mediated cell death. ANAP demonstrated direct cytolytic effects on NK/T lymphoma cells, inducing cytoskeleton-dependent and caspase- and complement-independent cell death via giant pore formation on the cell surface. This mechanism is similar to that of the mAb RE2 and mAb 4713 (Supplementary Fig. 1)^28–30^, and we have termed this antibody-induced cell death “anapocosis.”^28^.

Our findings showed that this newly established cytolytic antibody could recognize the ITGA4 (CD49d) molecule, which is an integrin alpha subunit that constitutes approximately half of the α4β1 lymphocyte homing receptor VLA-4. Another anti-ITGA4 antibody (natalizumab) is already used in clinical settings for the treatment of autoimmune diseases such as multiple sclerosis^32^; however, its therapeutic effect on malignant lymphoma and leukemia remains unknown. In this paper, we report the cytolytic activity of mAb ANAP against malignant lymphomas, which has not yet been observed with other ITGA4 antibodies.

## Results

### Cytolytic activity of mAb ANAP against NK/T-cell lymphoma cell lines

**Fig. 1:**
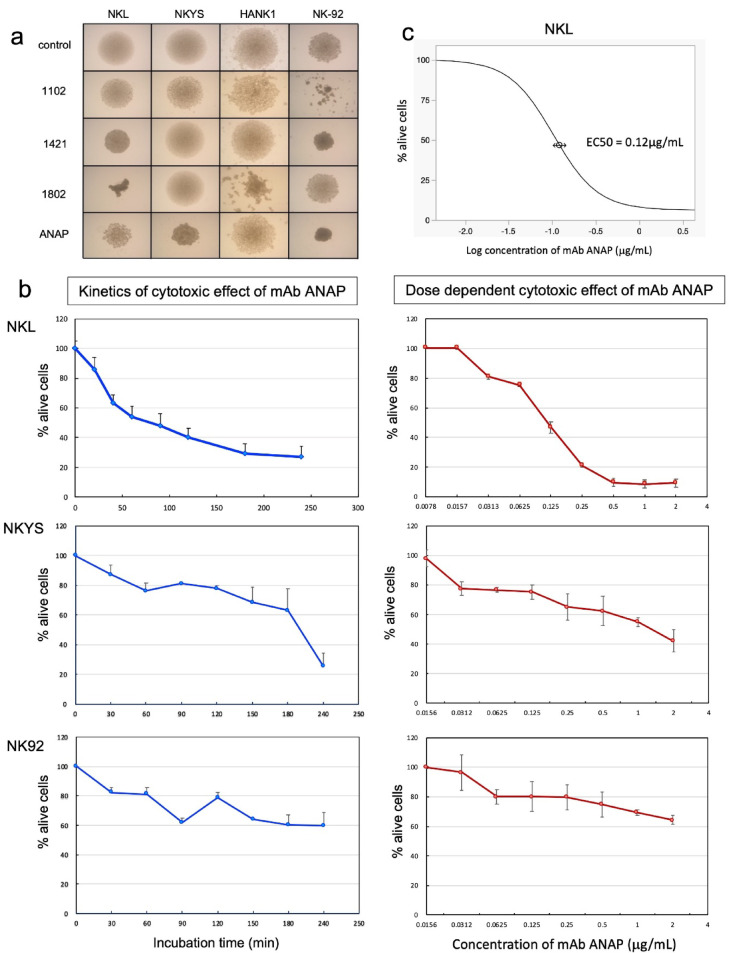
Profile of established mAbs. (**a**) Microscopic images of NK lymphoma cells after incubation with antibodies. Representative microscopic images of NK lymphoma cell lines treated with hybridoma culture supernatants. Control: culture medium alone. (**b**) Time- and dose-dependent cytotoxicity of mAb ANAP against NK/T lymphoma cell lines. Cells were treated with 3 µg/mL mAb ANAP for indicated times (left) or with indicated concentrations for 120 min (right). Viability was determined by dye exclusion test (mean ± SD, *n* = 3). (**c**) Dose-response curve of mAb ANAP against NKL cells. NKL cells were treated with various concentrations of monoclonal antibody ANAP, and cell viability was assessed. The percentage of alive cells was plotted against the logarithmic concentration of mAb ANAP (µg/mL). The half-maximal effective concentration (EC_50_) was determined to be 0.12 µg/mL, indicating the concentration required to achieve 50% cell death. Data points represent mean values.

Following our methodology, we obtained several mAbs, and hybridomas were screened by observing the morphological changes in NK/T-cell lymphoma cell lines after incubation with the culture supernatant of the hybridomas (Fig. 1a). ANAP, one of the cloned mAbs, directly induced rapid cell death in the NK/T-cell leukemia/lymphoma cell lines. Furthermore, mAb ANAP induced cell death in NK/T-cell leukemia/lymphoma cell lines in a time- and dose-dependent manner (Fig. 1b). For NKL cells, the EC_50_ value of mAb ANAP was determined to be 0.12 µg/mL (Fig. 1c).

**Fig. 2:**
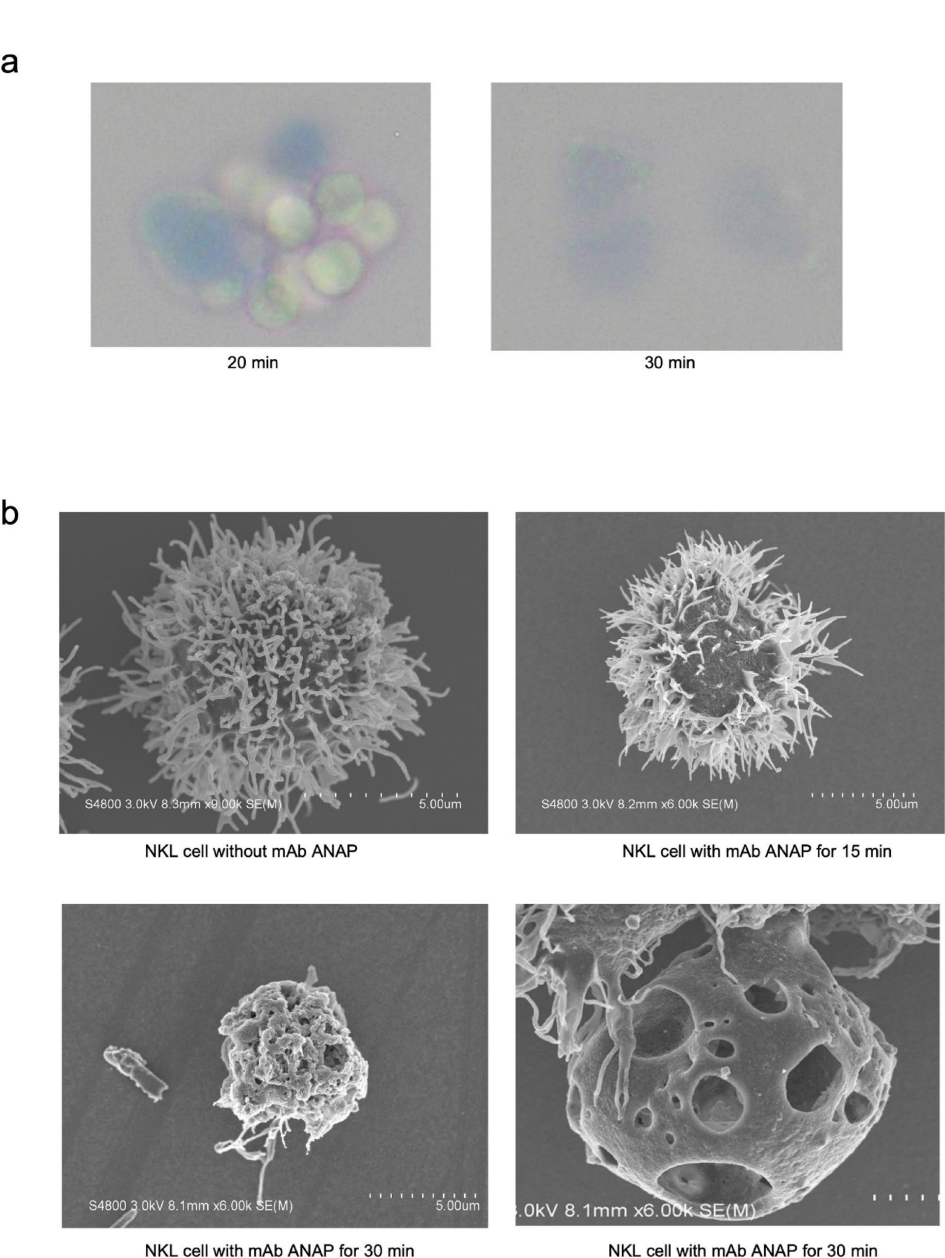
Microscopy-based analysis of mAb ANAP-induced cell death revealed the presence of giant pores. (**a**) A light microscopy image showing that mAb ANAP caused rapid aggregation and the death of individual NKL cells after 30 min, as evidenced by trypan blue exclusion staining. (**b**) Scanning electron microscopy image showing the formation of giant pores on NKL cells after 30 min incubation with mAb ANAP.

After 20 min of incubation with mAb ANAP, target NKL cells were observed as trypan blue-positive, either aggregated or dispersed, and light microscopy revealed that these cells dissolved and disappeared several minutes later (Fig. 2a). Almost all cells were completely lysed by mAb at the end of the experiments. Next, scanning electron microscopy confirmed this cell lysis and revealed the formation of giant pores on the surface of NKL cells during the early phase of mAb ANAP-induced cell lysis. Giant pores, approximately 3 μm in diameter, appeared on the NKL cell surface as early as 15 min after the addition of mAb ANAP to the cell suspension (Fig. 2b).

### Cytolytic activity of mAb ANAP against other NK or NK/T-cell leukemia/lymphoma cell lines

**Fig. 3:**
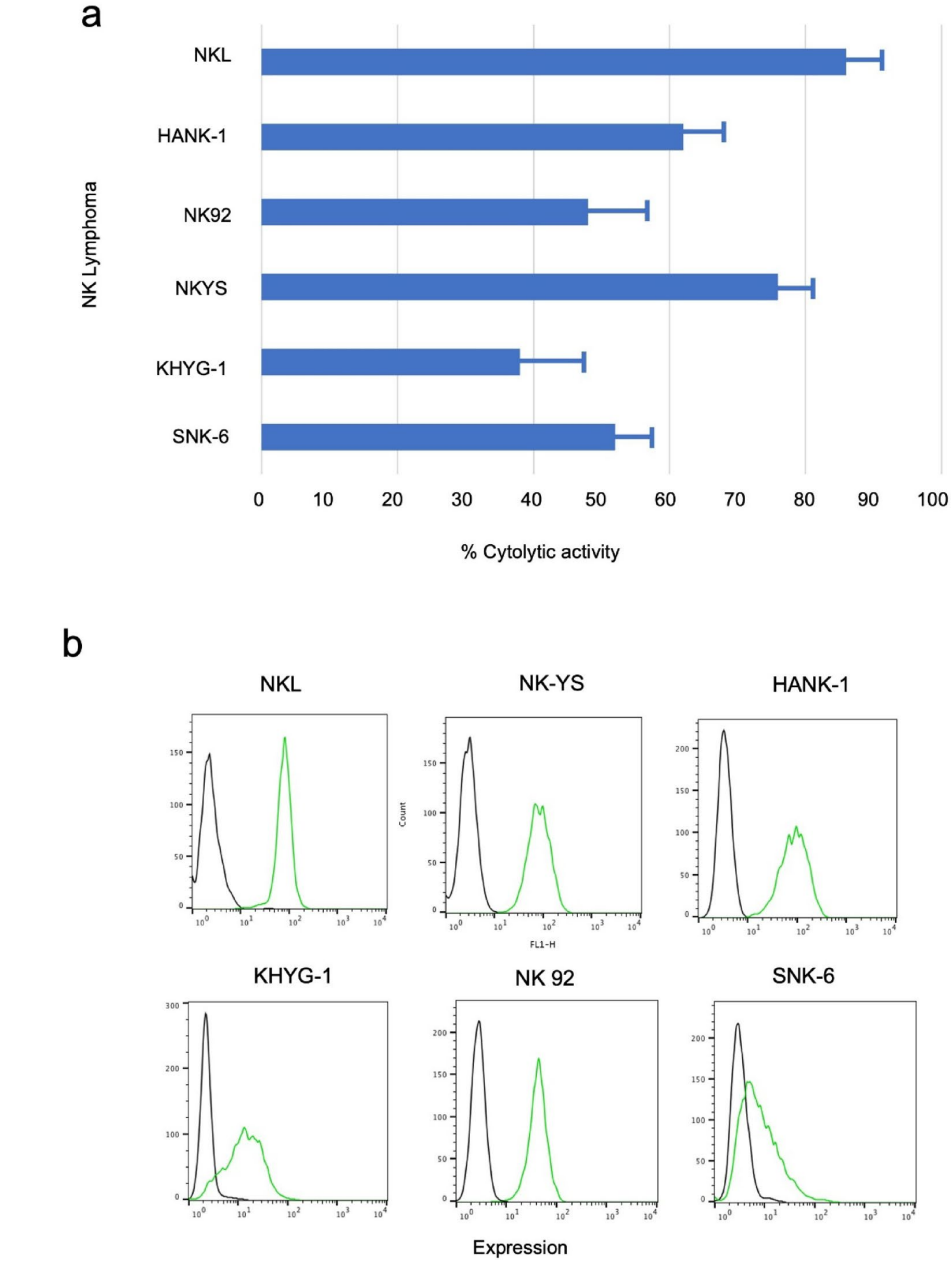
Reactivity and cytolytic activity of mAb ANAP against various lymphoma and leukemia cell lines. (**a**) The cytolytic activity of mAb ANAP was assessed by incubating a mixture of target cells (2 × 10^6^ cells/mL) in RPMI medium supplemented with 2% decomplemented (56 °C, 2 h) FCS and mAb (3 µg/mL) at 37 °C for 2 h. NK/T lymphoma/leukemia cell lines showed varying degrees of mortality. Approximately 30‒90% of the cells were killed within 2 h. (**b**) FCM analysis of mAb ANAP reactivity by incubating different lymphoma cell lines with mAb ANAP (4 °C, 30 min), followed by incubation with Alexa 488-conjugated rat anti-mouse Ig (green histogram).

We also tested the cytolytic activity of mAb ANAP on additional NK and NK/T-cell lines. The cells were incubated with mAb ANAP at 37 °C for 2 h, and we noted that this treatment induced rapid cell lysis in several NK and NK/T leukemia/lymphoma cell lines. Almost all of the tested cell lines showed varying degrees of mortality. NK lymphoma cell lines were killed within 2 h (Fig. 3a). We also found that the cytolytic activity was independent of serum complements, because cell death occurred even under serum-free culture conditions. Cell surface binding of mAb ANAP was confirmed by flow cytometry (FCM) (Fig. 3b).

### Lack of cytolytic activity of mAb ANAP on lymphocytes collected from healthy donors

**Fig. 4:**
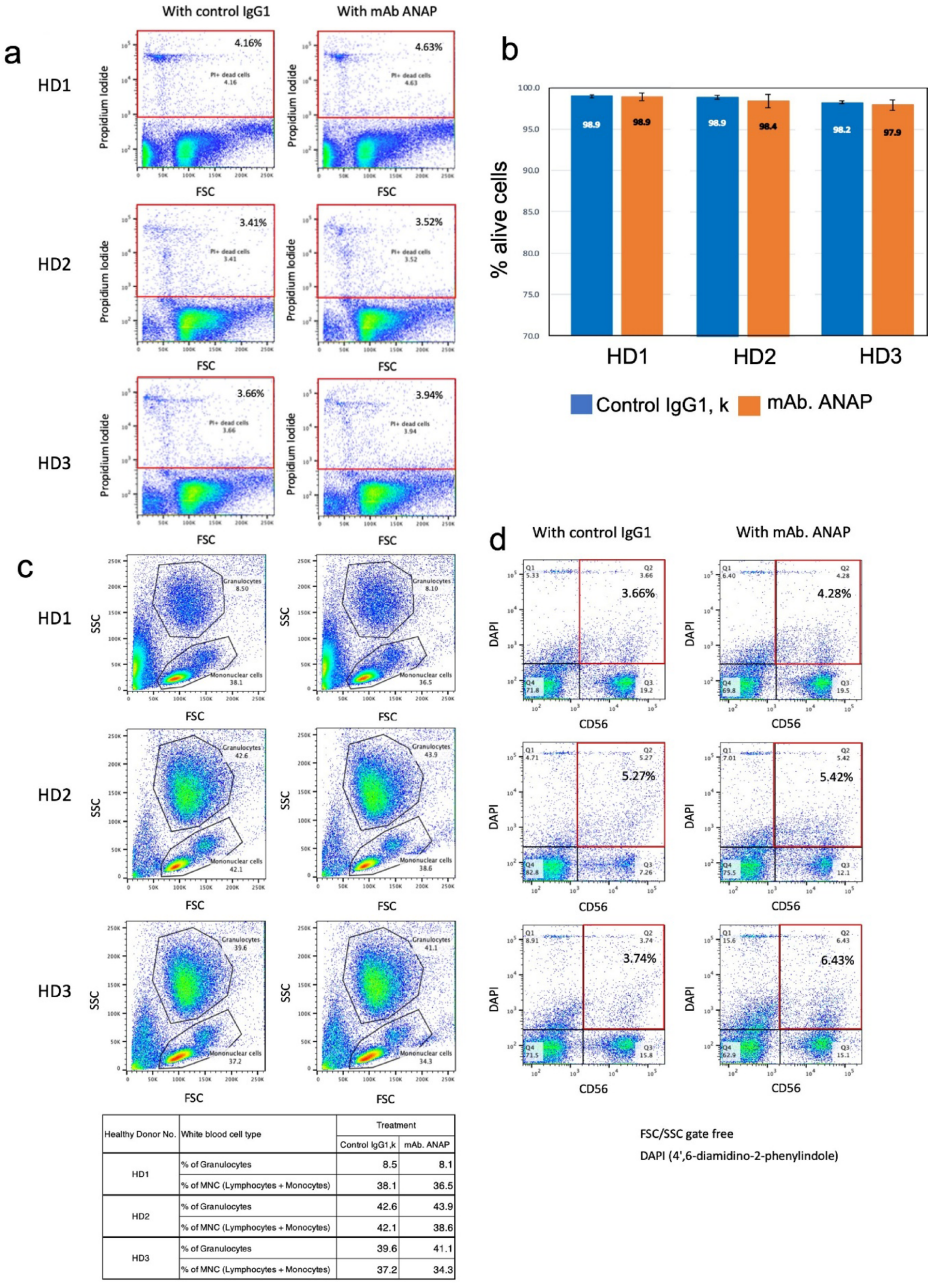
Lack of cytolytic activity of mAb ANAP against leukocytes from healthy donors. (**a**) Peripheral blood cells from three healthy donors (HD1-3) were treated with control IgG1 or mAb ANAP, followed by PI staining. Flow cytometry analysis data are shown, comparing the percentage of PI-positive dead cells. (**b**) PBMCs from three healthy donors (HD1-3) were treated with control IgG1 (blue bars) or mAb ANAP (orange bars), and viable cell percentages were calculated by dye exclusion assay and quantified in bar graphs. (**c**) FSC/SSC plots showing the distribution of granulocytes and mononuclear cells (MNC) by flow cytometry after peripheral blood cells from three healthy donors (HD1-3) were treated with control IgG1 or mAb ANAP. The table shows the percentages of granulocytes and mononuclear cells in each treatment. mAb ANAP did not significantly alter cell population distribution compared to the control antibody. (**d**) PBMCs from three healthy donors (HD1-3) were treated with control IgG1 (blue bars) or mAb ANAP (orange bars), followed by staining with anti-CD56 antibody and DAPI, and the percentage of CD56-positive dead cells was analyzed by flow cytometry. mAb ANAP did not induce cytotoxicity in CD56 + NK cells compared to the control antibody.

We examined the cytolytic activity of mAb ANAP against peripheral blood lymphocytes from three healthy donors. 2-hour incubation with mAb ANAP did not affect cell viability, and no cytolytic activity was observed against normal lymphocytes (Fig. 4a–c). To further confirm that mAb ANAP does not damage NK cells specifically, flow cytometry analysis was performed. The results showed that mAb ANAP treatment induced negligible cell death in CD56-positive NK cells from healthy donors (HD1-3), with DAPI-positive rates remaining comparable to those observed with control IgG1 (Fig. 4d).

### Candidate for the molecular target of mAb ANAP

**Fig. 5:**
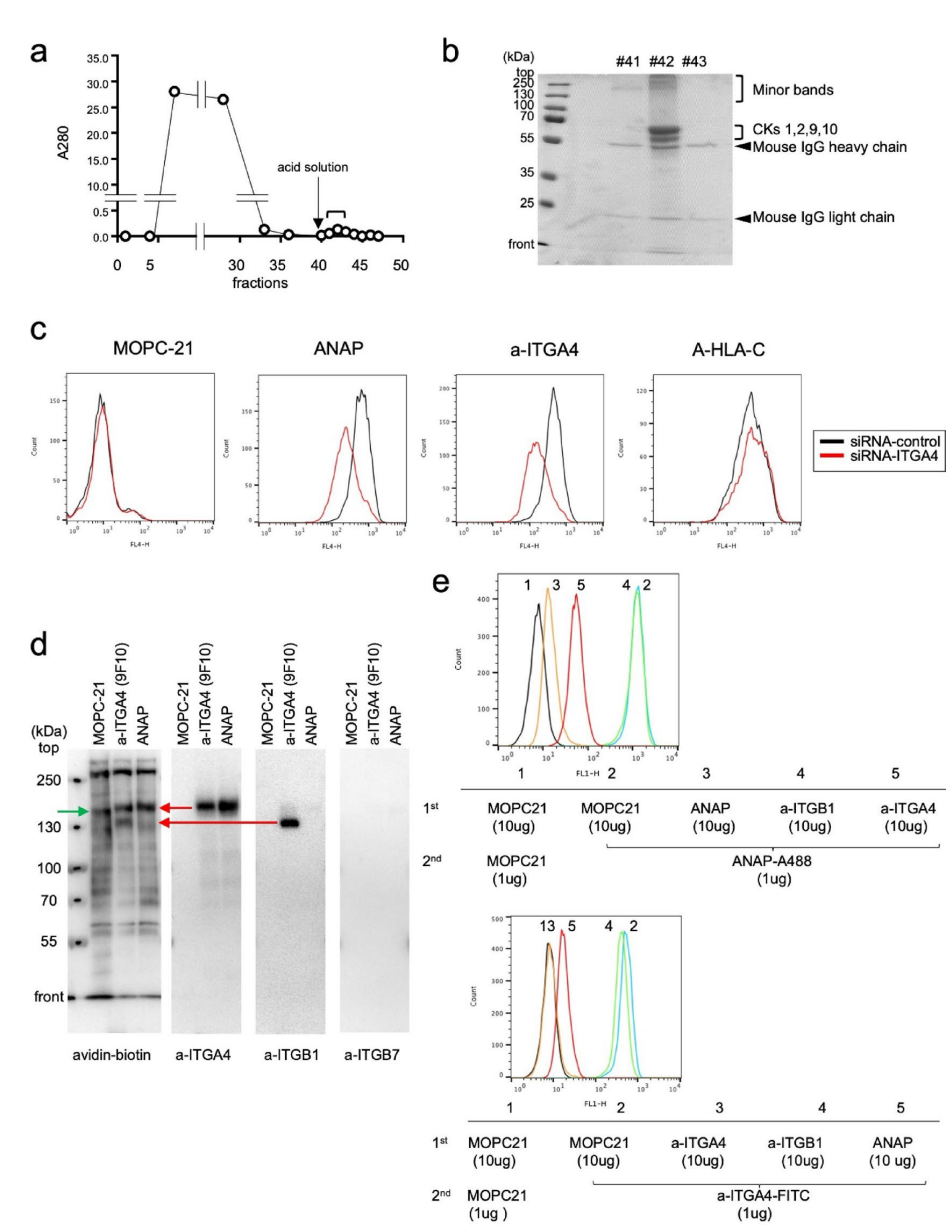
Identification of the molecular target of ANAP. (**a**) Affinity chromatography was performed using NKL cell surface membrane proteins extracted with 1% NP-40/PBS on an ANAP-conjugated HiTrap column (fraction: 1 mL). After washing, bound proteins were eluted with acidic solution from fraction #40 (arrow, 0.5 mL/fraction). (**b**) SDS-PAGE analysis of #41–43 fractions from (**a**). Protein bands were assigned based on LC-MS/MS analysis (Supplementary Table 2). (**c**) FCM analysis of MSTO-211 H cells transfected with control or ITGA4 siRNA. After 48 h, cells were analyzed using MOPC-21, α-HLA-C, α-ITGA4, and ANAP antibodies with anti-mouse IgG-Alexa 647 secondary antibody. (**d**) Biotin-labeled NKL cell lysates were immunoprecipitated with MOPC, α-ITGA4, or ANAP. Western blots were probed with streptavidin-peroxidase, then sequentially with α-ITGA4, α-ITGB1, and α-ITGB7 antibodies. (**e**) For epitope masking, NKL cells were pre-incubated with MOPC-21, α-ITGB1, α-ITGA4, or ANAP (10 µg), followed by ANAP-Alexa 488 (1 µg). Control used unlabeled MOPC-21. FCM analysis was performed, and the experiment was repeated using α-ITGA4-FITC as the labeled antibody.

The molecular target of mAb ANAP was identified using affinity chromatography with an mAb ANAP-coupled column. The elution profile showed a distinct peak (Fig. 5a), indicating successful protein recovery. SDS-PAGE analysis of the eluates revealed multiple protein bands (Fig. 5b). LC-MS/MS analysis identified 104 proteins (Supplementary Table 1), with the top 10 ranked proteins presented in Table 1. After excluding cytokeratins, ITGA4 emerged as the highest-ranking plasma membrane protein (rank 5), making it the most likely target of mAb ANAP.

**Table 1:** LC-MS/MS analysis of the eluate from affinity chromatography. 40 µL-aliquot of fraction #52 was pre-treated appropriately and subjected to LC-MS/MS analysis.

No	Uused	Total	% Cov	% Cov (95)	Accession #	Name
1	117.49	117.49	93.3	89.7	sp|P35908|K22E_HUMAN	Keratin, type II cytoskeletal 2 epidermal OS = Homo sapiens OX = 9606 GN = KRT2 PE = 1 SV = 2
2	105.38	116.66	81.8	74.8	sp|P04264|K2C1_HUMAN	Keratin, type II cytoskeletal 1 OS = Homo sapiens OX = 9606 GN = KRT1 PE = 1 SV = 6
3	87.1	87.1	83.3	83	sp|P35527|K1C9_HUMAN	Keratin, type I cytoskeletal 9 OS = Homo sapiens OX = 9606 GN = KRT9 PE = 1 SV = 3
4	80.17	81.55	69.4	65.1	sp|P13645|K1C10_HUMAN	Keratin, type I cytoskeletal 10 OS = Homo sapiens OX = 9606 GN = KRT10 PE = 1 SV = 6
5	68.82	68.82	55.8	46.9	sp|P13612|ITA4_HUMAN	Integrin alpha-4 OS = Homo sapiens OX = 9606 GN = ITGA4 PE = 1 SV = 3
6	48.43	61.71	63.6	45.9	sp|P13647|K2C5_HUMAN	Keratin, type II cytoskeletal 5 OS = Homo sapiens OX = 9606 GN = KRT5 PE = 1 SV = 3
7	47.97	56.03	63.6	56.1	sp|P02533|K1C14_HUMAN	Keratin, type I cytoskeletal 14 OS = Homo sapiens OX = 9606 GN = KRT14 PE = 1 SV = 4
8	41.14	41.13	15.8	11	sp|P15924|DESP_HUMAN	Desmoplakin OS = Homo sapiens OX = 9606 GN = DSP PE = 1 SV = 3
9	34.84	34.84	26.7	26.7	sp|P05556|ITB1_HUMAN	Integrin beta-1 OS = Homo sapiens OX = 9606 GN = ITGB1 PE = 1 SV = 2
10	26.33	26.33	55.5	48.6	sp|P06733|ENOA_HUMAN	Alpha-enolase OS = Homo sapiens OX = 9606 GN = ENO1 PE = 1 SV = 2

### Flow cytometry of SiRNA transfected cells

To validate ITGA4 as ANAP’s molecular target, we performed siRNA knockdown experiments in MSTO-211 H cells. Flow cytometry analysis revealed that ITGA4 siRNA transfection resulted in concurrent downregulation of both ITGA4 and the ANAP-target (Fig. 5c). Knockdown of higher-ranked proteins (CKs1, 2, 9, and 10) or had no effect on ANAP-target expression (Supplementary Figs. 2 and 3). These results strongly suggested ITGA4 or its associated integrin beta-chain as ANAP’s molecular target.

### Side-by-side comparison of immunoprecipitates captured by ANAP and anti-ITGA4

Side-by-side comparison of immunoprecipitates from NKL cell lysates was performed using ANAP and commercial anti-ITGA4 (9F10) antibodies. Western blot analysis revealed ~ 150 kDa biotinylated bands in both 9F10- and ANAP-lanes, while a unique ~ 130 kDa band appeared only in the 9F10-lane (Fig. 5d, leftmost panels). Rehybridization identified the ~ 150 kDa protein as ITGA4 in both lanes, and the ~ 130 kDa protein as ITGB1 exclusively in the 9F10-lane (Fig. 5d, middle panels). While ITGA4 typically forms heterodimers with ITGB1 (VLA-4) or ITGB7 (LPAM-1), the ANAP-lane showed only ITGA4, with neither ITGB1 nor ITGB7 detected (Fig. 5d, middle to rightmost panels; Supplementary Figs. 4, 5).

These results suggest ANAP binds specifically to the ITGA4 moiety of VLA-4, potentially inducing a conformational change that releases ITGB1 from the heterodimer. The detection of ITGB1 in previous affinity chromatography (rank 9, Table 1) likely resulted from different washing conditions (14-fold lower buffer volume) and the higher sensitivity of LC-MS/MS compared to immunoblotting.

### Measurement of the epitope masking effect

To confirm that ITGA4 is the target of mAb ANAP, we measured the “epitope masking effect”^29^ of two anti-ITGA4 antibodies, ANAP and 9F10. NKL cells were first incubated with a control antibody, MOPC-21, followed by the addition of mAb ANAP labeled with Alexa Fluor 488 (Fig. 5e, upper panel). Since both epitopes target distinct molecules on the cells, the labeled mAb ANAP could independently bind to the cells, resulting in strong FCM signal emission (Fig. 5e, upper panel).

In contrast, initial incubation of the NKL cells with anti-ITGA4 (9F10) and subsequent addition of the mAb ANAP-Alexa Flour 488 induce a distinct reduction in the binding ability of ANAP-Alexa Flour 488, as shown by the significant downward shift of the signal peak in FCM (Fig. 5e, upper panel).

These results indicate that the epitopes of both antibodies overlap on a single molecule, i.e., ITGA4. The same conclusion was reached by the results of a reverse experiment in which NKL cells were first incubated with ANAP, followed by the addition of labeled anti-ITGA4(9F10)-FITC (Fig. 5e, lower panel).

### mAb ANAP, but not other anti-ITGA4 antibodies, induces direct cell death in NK lymphoma cells

The mAb ANAP developed in the present study induces complement-, ADCC-, and ADCP-independent direct cell death in NK/T lymphoma cells. In contrast, both natalizumab and the well-known anti-ITGA4 mAb 9F10 did not exhibit killing activity against NK lymphoma cell lines (Supplementary Fig. 6).

### Expression of ANAP on the peripheral blood of healthy donors

**Fig. 6:**
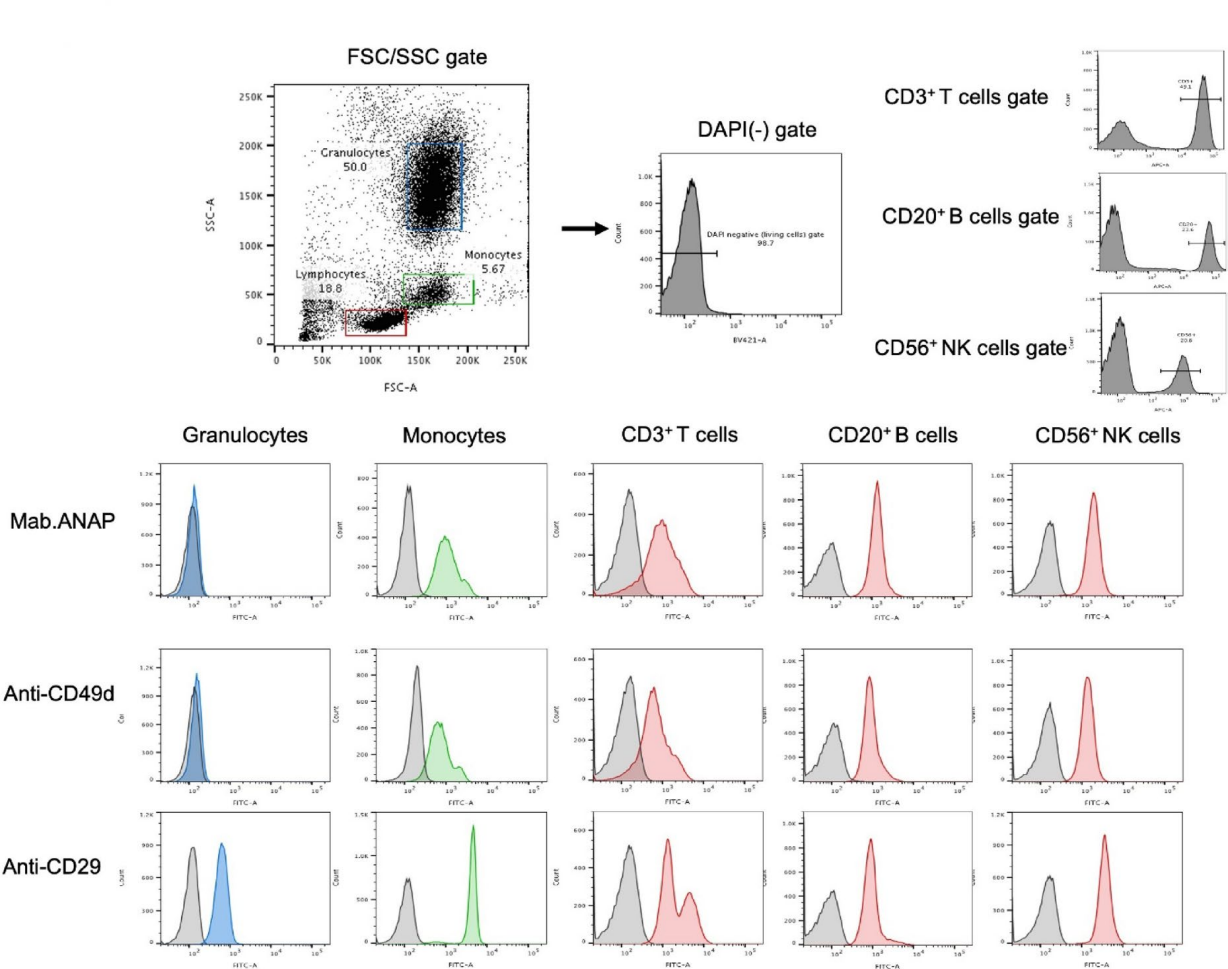
Expression of ANAP on the peripheral blood of healthy donors. We used FSC and SSC to determine granulocytes, monocytes, and lymphocytes, and lymphocytes were further categorized into T, B, and NK cells using anti-CD3, CD20, and CD56 markers, respectively. Cells were incubated with anti-CD49d (9F10) and anti-CD29 for 30 min and then with Alexa 488-conjugated anti-mouse IgG. Negative controls are represented by black lines. The mAb ANAP showed the same FACS profiles as the existing antibody did against anti-CD49d (ITGA4).

ITGA4 is known to be expressed on lymphocytes and monocytes, but not on neutrophils. In contrast, Integrin β1 is known to be expressed on neutrophils. When staining normal human peripheral blood with the ANAP antibody, our results confirmed that the observed reactivity was similar to that of the authentic anti-ITGA4/CD49d (HP2/1) antibody (Fig. 6).

### Testing the effects of several potential inhibitors to investigate the mechanism underlying mAb ANAP-induced cell death

**Fig. 7:**
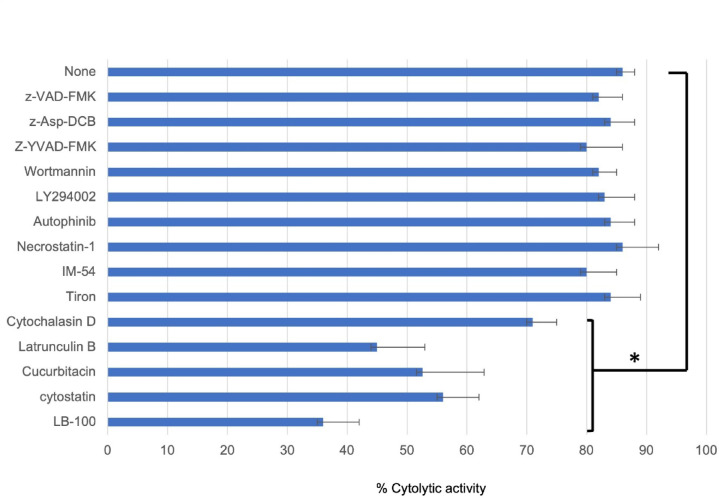
The mechanism of mAb ANAP-induced cell death against NKL cells involving the cytoskeleton and PPA2. The inhibitors were added 1–2 h before the cytotoxicity assay, and the percentage of dead cells was determined by trypan blue dye exclusion. The agents were caspase inhibitors z-VAD-FMK and z-Asp-DCB, pyrotosis inhibitor Z-YVAD-FMK, PI-3 kinase inhibitors, wortmannin and LY294002, autophagy inhibitor autophinib, necroptosis inhibitor necrostatin-1, necrosis inhibitor IM-54, cytoskeletal inhibitors cytochalasin D and latrunculin B, reactive oxygen species scavenger Tiron, PP2A inhibitors cytostatin, and LB-100. *p = 0.05 vs. None treated. Each value represents mean ± SD.

To investigate the mechanism underlying mAb ANAP-induced cell death, we tested the effects of various potential inhibitors on the cytolytic activity of mAb ANAP against NKL cells. Our results revealed that the caspase inhibitors z-VAD-FMK^33^ and z-Asp-DCB^34^, the caspase and pyroptosis inhibitor Z-YVAD-FMK^35^, the PI-3 kinase inhibitors wortmannin^36^ and LY294002^37^, and the autophagy inhibitor Autophinib^38^ did not inhibit the cytolytic activity of mAb ANAP (Fig. 7). To further differentiate between mAb ANAP-induced cell death and apoptosis, we performed additional experiments as follows. Following treatment with anti-Fas mAb or mAb ANAP, the target cells (Jurkat and NKL) were stained with cleaved caspase-3 (Asp175)-specific antibody (Cell Signaling) and analyzed by FCM. Although treatment of Jurkat cells with anti-Fas antibody resulted in caspase 3 activation and subsequent apoptosis, treatment with mAb ANAP did not activate caspase 3, indicating that mAb ANAP did not induce apoptosis-related cell death (Supplementary Fig. 7). Caspase-3-independent cytotoxicity of mAb ANAP. Furthermore, the necroptosis inhibitor necrostatin-1^39^ and the necrosis inhibitor IM-54^40^ also failed to inhibit the cytotoxicity of mAb ANAP (Fig. 7), thereby suggesting that mAb ANAP-induced cell death is not related to apoptosis, necroptosis, or necrosis.

Inhibition tests using caspase-3 and autophagy inhibitors did not indicate the involvement of pyroptosis or autophagy in mAb ANAP-induced cell death. Additionally, a cell-permeable reactive oxygen species (ROS) scavenger, 4,5-dihydroxy-1, 3-benzene sulfonic acid (Tiron)^41^, had no effect on mAb ANAP-induced cell death. In addition, we did not observe any ROS production after exposure to mAb ANAP (Supplementary Fig. 7), indicating that ROS, which are associated with pyroptosis and autophagy, are not involved in ANAP-induced cell death.

In contrast, both cytochalasin D, which depolymerizes cytoskeletal actin filaments to actin monomers^42,43^, and latrunculin B, which reduces the monomeric actin pool available for polymerization^43,44^, inhibited mAb ANAP-induced cell death (Fig. 7). These results are consistent with the findings of previous studies regarding the cytolytic activity of anti-pan MHC class I mAb and anti-pan HLA class II mAb against lymphocytes and lymphoma cells^28–30^. Therefore, it can be inferred that cytolytic mAb ANAP and anti-pan MHC class I and II antibodies may employ common signaling events to induce cell death.

Additionally, we used the Screening Committee of Anticancer Drugs (SCADS) inhibitor kit (400) (https://www.molpro.jp/explore/library/#hyoujun)^45^ to identify signaling pathways involved in mAb ANAP-induced killing. In addition to the expected inhibitors (cytochalasin, latrunculin), we found that cucurbitacin I^46^ and Cytostatin^47^ and LB-100^48^ phosphatase AII inhibitors could partially, yet firmly, block the cytolytic effect of mAb ANAP on NKL cells. These results suggest the existence of a new signaling pathway involved in antibody-induced killing.

## Discussion

A common strategy for developing mAbs targeting lymphoma cells is to identify cell surface antigens that are preferentially expressed on lymphoma cells rather than normal lymphocytes^49^. In recent years, several antigens have been identified, including CD25, CD32, CD44, CD95, CD123, and CLL-1, and, as a result, mAbs targeting these molecules have been developed^50,51^. Additionally, monoclonal antibodies targeting CD44, CD47, and CD123 have shown efficacy against lymphoma in xenograft models^50,51^. The greatest advantage of immunizing mice with whole tumor cells is that this approach preserves the potential of generating novel antibodies against unknown molecules that are preferentially expressed on tumor cell surfaces. Therefore, in the present study, we used novel immunization and screening methods to generate therapeutic mAbs for NK/T lymphoma and leukemia cell lines against molecules that were not previously considered therapeutic targets.

BALB/c mice were alternately immunized with cells from two NK/T lymphoma cell lines, and hybridoma clones were screened for direct cytolytic effect on NK/T leukemia cell lines that were not used for immunization. This strategy aimed to obtain mAbs with damaging activity against not a single but several NK/T lymphoma/leukemia cell lines. In the first screening, we used light microscopy and trypan blue to identify whether hybridoma supernatants could directly kill the target cells. This approach negated the need to perform ELISA assays or FCM analysis, thus saving considerable labor during this process. Our experiments resulted in the identification of a mAb ANAP with direct cytolytic activity against NK lymphoma and leukemia cells. Notably, NK-YS, KHYG-1 and SNK-6, all cell lines of ENKL, were also susceptible to the cytolytic effect of mAb ANAP.

We previously reported RE2 (anti-pan MHC class I) and 4713 (anti-pan HLA class II) monoclonal antibodies that induce complement-, ADCC-, and ADCP-independent direct cell death in malignant lymphoma cells. The mAb ANAP developed in the present study similarly induced direct cell death. Natalizumab, an anti-ITGA4 antibody, is currently used in clinical settings for treating multiple sclerosis^32^. However, unlike mAb ANAP, neither natalizumab nor the well-known anti-ITGA4 mAb 9F10 exhibited killing activity against NK lymphoma cell lines, suggesting that the cytotoxic effect of mAb ANAP may depend on recognition of a distinct epitope on ITGA4. We have established mAbs that rapidly induce the formation of giant pores on the surface of lymphoma/leukemia cells (Fig. 2, Supplementary Fig. 1)^28–30^. The diameter of the pores induced by perforin-producing CD8 cytotoxic T cells or complement C9 activated with Abs was approximately 0.02 μm^52,53^. In contrast, the diameter of the pores induced by these mAbs was more than 100 times larger than that induced by perforin or complement C9. We named this type of cell death “anapocosis” (not-apoptosis) after the Japanese word for “hole”^28^. Furthermore, our results showed that mAb ANAP kills NK lymphoma cells by creating holes of a similar size to those formed by previously established anapocosis antibodies, suggesting that the same death signals are involved in this process.

Anapocosis is characterized by the generation of large holes (approximately 3 μm) in the cell membrane. Despite some morphological similarities, pyroptosis inhibitors did not inhibit anapocosis-induced cell death (Fig. 7). The cytolytic activity of anti-MHC mAbs (RE2, 4713) and mAb ANAP was considerably blocked by latrunculin B and cytochalasin D (Fig. 7). Furthermore, cytostatin and cucurbitacin, which are phosphatase inhibitors blocking cell-cell adhesion, also managed to reduce cytolytic activity significantly. Obinutuzumab, used in the treatment of CD20-positive follicular lymphoma, causes direct cell death in lymphoma cells as a result of cell-to-cell adhesion^54^. Our findings suggest that the ANAP antibody may share some of the cell death mechanisms with obinutuzumab. However, cell-cell adhesion between target cells is considered essential for obinutuzumab-induced cell death, whereas cell-cell adhesion between target cells is common with anapocosis antibodies, but not necessarily required^28^.

Although the cytolytic activity of the anapocosis antibodies (anti-MHC mAbs RE2 and 4713, and mAb ANAP) was blocked by latrunculin B and cytochalasin D, Fab fragment of these mAbs showed no cytolytic effects. These results suggest that both the cytoskeleton and the crosslinking of target molecules are essential for anapocosis. Although several antibodies inducing the direct death of target cells in an ADCC- or complement-independent manner are already known, many forms of cell death differ from typical apoptosis and necrosis^25^. Necroptosis, for example, involves the loss of cell membrane integrity due to the phosphorylation of the pseudo-kinase mixed lineage kinase domain-like protein (MLKL) by receptor-interacting protein kinase 3 (RIPK3)^55–57^. RIPK3, MLKL, and ZBP1 can regulate inflammasomes and pyroptosis^55–60^. Moreover, RIPK1 is widely recognized as a key regulator downstream of TNFR1 activation, influencing NF-κB activation and determining whether cells undergo apoptosis, necroptosis, or pyroptosis^57,59,60^. As the RIPK1 inhibitor Necrostatin-1 also failed to inhibit ANAP-induced cell death, the mechanism of cell death induced by mAb ANAP appears to be different from both necroptosis and pyroptosis^61^.

In addition to cytochalasin D and lutrunclin B, cucurbitacin blocked the cytolytic activity of mAb ANAP. Cucurbitacin I inhibits the actin depolymerization protein cofilin I, thereby inhibiting the depolymerization of F-actin^46,62^. In other words, cucurbitacin may act as an inhibitor of actin depolymerization. It has been reported that the anapocosis antibody mAb 4713 induces uneven accumulation of actin filaments on the cell surface, and that this killing activity can be blocked by actin polymerization inhibitors, such as cytochalasin and latrunculin^30^. Since actin undergoes reversible polymerization and depolymerization, we hypothesized that actin depolymerization inhibitors may also inhibit the efficacy of the anapocosis antibodies by preventing actin from working properly. Although cucurbitacin is known as a JAK-2 inhibitor^63^, current literature suggests that it is not very selective.

PP2A inhibitors, including cytostatin and LB-100, also blocked the cytolytic effect of ANAP. Cytostatin is known as a cell adhesion inhibitor that disrupts intracellular signals related to cell adhesion by inhibiting the phosphorylation of FAK and paxillin^46^. Cytostatin also inhibits serine/threonine dephosphorylation by selectively inhibiting PP2A^61^, which results in enhanced serine/threonine phosphorylation. A previous study showed that cytostatin might modify paxillin through serine-threonine phosphorylation to inhibit cell adhesion^46^. If cell adhesion factors, as well as actin reorganization, are involved in the cell aggregation induced by the ANAP antibody, inhibition of these processes may impair aggregation and consequently reduce the efficacy of ANAP. While these findings support our results noted with cytochalasin and latrunculin, further research is needed to elucidate the events occurring post-aggregation based on these results alone. Additionally, protein phosphatases are important kinases involved in cytoskeleton formation and negative regulators of signaling cascades. Despite its historical role as a tumor suppressor, inhibition of PP2A paradoxically presents a potential therapeutic strategy for various types of cancers^61,64,65^. For example, LB100, a water-soluble small molecule PP2A competitive inhibitor, has been reported to be effective in the treatment of ovarian cancer, myelodysplastic syndrome, and relapsed solid tumors^66^.

We have conducted extensive mechanistic analyses; however, several aspects remain unclear. Although all anapocosis antibodies, including ANAP, mAb 4713, and mAb RE2, selectively kill cancer cells while sparing normal cells, we have not yet elucidated why these antibodies bind to normal cells without killing them, despite investigating numerous hypotheses. Additionally, it remains unclear why mAb 4713, despite binding strongly to NK/T lymphoma cells, fails to kill them. In this study, we established ANAP antibody, which can kill NK/T lymphoma cells that were resistant to mAb 4713; however, we were unable to clarify why the ability to bind does not equate to the ability to induce cell death. Understanding the mechanism underlying the selective cytotoxicity of anapocosis antibodies toward cancer cells remains an important question for future investigation.

Given the promising cytolytic activity of mAb ANAP against NK lymphoma cells, we are actively pursuing its clinical development as a therapeutic agent. Notably, mAb ANAP demonstrated remarkably potent activity, with an EC_50_ value of 0.12 µg/mL for NKL cells (Fig. 1c). This value is substantially lower than that reported for obinutuzumab (GAZYVA), which exhibits an EC_50_ range of approximately 19.1–73.5 µg/mL for direct cell death induction^67^, suggesting superior potency of mAb ANAP. To evaluate its in vivo efficacy and safety profile, we have established xenograft models using NK lymphoma cell lines and are conducting preclinical studies to assess its therapeutic potential in these animal models. Importantly, anapocosis-inducing mAbs, including mAb ANAP, exhibit no direct cytotoxicity toward normal cells; mAb ANAP does not kill normal lymphocytes expressing ITGA4, demonstrating remarkable selectivity for malignant cells. However, the potential for damage to normal cells via CDC, ADCC, and ADCP mechanisms cannot be entirely excluded. To address this concern, we are currently generating a humanized version of mAb ANAP with modifications to the Fc region designed to minimize effector functions and prevent potential damage to endothelial cells and normal lymphocytes. These strategic modifications aim to preserve the direct cytolytic activity of mAb ANAP while reducing the risk of off-target toxicity. These comprehensive efforts represent critical steps toward translating our findings into a clinically viable and safe immunotherapy for patients with NK/T-cell lymphomas, particularly those who are refractory to conventional treatments or are immunocompromised.

## Materials and methods

### Ethics

This study has been performed according to the principles of the 1964 Helsinki declaration and approved by the Ethical committee at Juntendo University School of Medicine, Tokyo, Japan (Permission ID number 14–95). The study is conducted in accordance with ARRIVE guidelines.

*SCADS library* (https://www.molpro.jp/explore/library/#hyoujun). SCADS library was kindly provided by the Screening Committee of Anti-cancer Drugs supported by Grant-in-Aid for Scientific Research on Priority Area “Cancer” from the Ministry of Education, Culture, Sports, Science and Technology, Japan.

### Mice and cells

BALB/c and mice at 8 weeks of age were obtained from Japan SLC Inc. (Hamamatsu, Japan). All mice were maintained under specific pathogen-free conditions. The Ethics Review Committee for Animal Experimentation of Juntendo University Faculty of Medicine approved all animal experiments (Project Number 727). Mice were euthanized using sodium pentobarbital, and measures were taken to minimize suffering. Peripheral mononuclear cells were collected from healthy adult volunteers. Informed written consent was obtained from all study participants. Peripheral blood lymphocytes were isolated from consenting healthy blood donors via centrifugation over Histopaque (Sigma Chemical Co., St. Louis, MO). Human NK/T-cell lymphoma cell lines (NKL, NK-YS, HANK-1, NK-92, KHYG-1, and SNK-6) were purchased from the American Type Culture Collection (Manassas, VA), the German Collection of Microorganisms and Cell Cultures and DSMZ (Braunschweig, Germany), or the Japan Cancer Research Resources Bank (Osaka, Japan). The cell lines were cultured in RPMI 1640, DMEM or IMDM medium containing 10–15% heat-inactivated fetal calf serum (FCS). For all NK/T-cell lymphoma cell lines, 7.5 ng/ml of IL-2 (PeproTech EC; London, UK) was further added to the culture medium.

### Reagents and antibodies

Latrunculin B, Protein Phosphatase 2 A (PP2A) inhibitors, Cytostatin, Cantharidin, and LB-100 were purchased from Abcam (Cambridge, UK). Z-YVAD-FMK, Z-VAD-FMK, and Z-Asp-DCB were purchased from the Peptide Institute Inc. (Osaka, Japan), and cathepsin inhibitor III (Z-FG-HNO-BzOME) from Calbiochem (Tokyo, Japan). IM-54 was purchased from Santa Cruz Biotechnology (UK). Necrostatin-1 was purchased from Enzo Biochem Inc. (NY). EDTA, forskolin, cytochalasin D, 4,5-dihydroxy-1, and 3-benzene sulfonic acid (Tiron) were purchased from Sigma-Aldrich. Autophinib was purchased from Selleckchem (Houston TX). Ly294002, PI3Kinase inhibitor, and autophagy inhibitor were purchased from FUJIFILM Wako (Osaka, Japan). Actin polymerization inhibitor, cytochalasin D and Alexa 488-conjugated rat anti-mouse Ig, mouse IgG, propidium iodide, and DAPI (4’,6-diamidino-2-phenylindole) were purchased from Sigma-Aldrich. MOPC-21mouse IgG1, and kappa monoclonal isotype control Ab were purchased from Abcam (Cambridge, UK). Alexa Fluor 647-conjugated anti-human CD3 (UCHT1), anti-human CD20 (2H7), anti-human CD56 (5.1H11), and Anti-ITGA4 (9F10)-FITC antibodies were purchased from BioLegend (San Diego, CA). Anti-Fas mAb was purchased from MBL (Nagoya, Japan). Alexa 488-conjugated goat anti-rabbit IgG (H + L) antibody was purchased from Thermo fisher Science (MA). Integrin alpha 4 (ITGA4)/CD49d antibody (HP2/1) was purchased from FUJIFILM Wako (Osaka, Japan). Natalizumab was purchased from Eisai Co., Ltd. (Tokyo, Japan). Goat anti-Mouse IgG (H + L) Cross-Adsorbed Secondary Antibody, Alexa Fluor™ 647 Invitrogen (MA, USA). Purified mouse anti-human CD29 (ITGB1) Antibody clone: TS2/16, mouse anti-human Integrin beta 7 (ITGB7) antibody clone: 473,207, purified mouse anti-human HLA-A, B,C (MHC class I) antibody clone: W6/32, purified mouse anti-human HLA-C antibody clone: DT-9 was purchased from BioLegend (San Diego, CA). Mouse anti-human Integrin beta 7 (ITGB7) antibody clone: 473,207 was purchased from R&D Systems (Minneapolis MN).

### Establishment of mAb ANAP

BALB/c mice were immunized intraperitoneally every two weeks for four months using live NK/T-cell lymphoma cells. The injections alternated between NK-YS and HANK-1 cells to establish mAbs reactive to common molecules on the surface of NK cell lines. Three days after the last immunization, mouse spleen cells were fused with P3U1 non-producing myeloma cells using the polyethylene glycol method, and hybridomas were selected using hypoxanthine-aminopterin-thymidine culture medium (Corning Cellgro; VA, USA). Hybridomas producing antibodies with direct cytolytic activity against a third NK lymphoma cell line, namely NKL, were selected based on the trypan blue dye exclusion test and confirmed by flow cytometry analysis. This method was used to develop mAbs with direct cytolytic effects on multiple NK/T-cell lymphoma cell lines and to avoid generating clonotypic mAbs specific to a single cell line. We identified one mAb, named mAb ANAP, which consistently induced direct cell death in NK/T-cell lymphoma cell lines.

### Flow cytometry analysis

Live NK/T-cell lymphoma cells (2 × 10^5^ cells) were washed in PBS containing 1% FBS, 1 mM EDTA, and 0.1% sodium azide, incubated with 100 µl of hybridoma supernatant or 3 µg/ml of mouse IgG1, kappa monoclonal isotype control Ab (MOPC-21) for 25 min at 4 °C. Cells were subsequently stained with Alexa 488-conjugated gout anti-mouse Ig G for 25 min at 4 °C. Fluorescence intensity was measured with a BD LSRFortesa cell analyzer (BD Biosciences, San Diego, CA, USA), and data were analyzed by Flow Jo software (Flow Jo 10.4, LLC, Ashland, OR, USA).

### Cytolytic assay

We assessed the cytolytic activity of mAb ANAP by incubating a mixture of target cells (2 × 10^6^ cells/ml) in RPMI medium supplemented with 2% decomplemented (56 °C, 30 min) FCS and mAb (0.3 µg/ml) at 37 °C for 2 h. Cell lysis was determined in triplicate using trypan blue dye exclusion, and the percentage of lysed cells was calculated.

Potential inhibitors for the cytolytic activity of mAb ANAP to NKL cells, Z-YVAD-FMK, z-VAD-FMK, z-Asp-DCB, LY294002, latrunculin B and cucurbitacin were added in cell cultures 1 h before, while wortmannin and cytostatin and cucurbitacin were added 2 h and 24 h before the addition of mAb ANAP. Sodium azide, EDTA, and cytochalasin D were added to the assay medium during the cytolytic assay to test the effects of these reagents. Reagent concentrations were optimized based on preliminary experiments described previously^28–30^. Cellular ROS were observed using the DCFDA-Cellular ROS Detecting Assay Kit purchased from Abcam.

### Affinity chromatography

Purified mAb ANAP (10 mg) was immobilized on HiTrap NHS-activated HP (1 mL; Cytiva) according to manufacturer’s protocol. NKL cells (3 × 10^9^) were lysed overnight at 4 °C in PBS (pH 7.0) containing 1% NP-40 and protease inhibitors. The clarified lysate was applied to the ANAP column, washed with extraction buffer, and bound proteins were eluted with 0.1 M glycine-HCl (pH 2.7) in 0.5 mL fractions. Eluates were neutralized with 1 M Na2HPO4 and analyzed by 11% SDS-PAGE^68^. Samples were heat-denatured in SDS-PAGE buffer before electrophoresis, and gels were stained with Coomassie blue R-250.

### LC-MS/MS

Proteins were identified by nano-LC-MS/MS using a Triple TOF 5600 system operated with the Analyst TF 1.7 software and an Eksigent nano-LC system (AB SCIEX, Framingham, MA, USA). The obtained MS data was searched with ProteinPilot 5.0.1 Software (AB SCIEX) using the UniProt database (2018_11). A confidence cutoff of 1% false discovery rate was applied for protein identification^69^.

### Small interfering RNA (siRNA) transfection

MSTO-211 H cells were transfected in 60-mm plates at 60–70% confluency using Lipofectamine RNAiMAX (Thermo Fisher Scientific). For target knockdown, cells received a mixture of five siRNAs (100 pmol each, sequences in Supplementary Table 2a) in 400 µL Opti-MEM I. Control transfections used three non-targeting siRNAs (167 pmol each, Supplementary Table 2b). After 4 h, complete medium was added and cells were cultured for 48 h. Knockdown efficiency was analyzed by flow cytometry (FACSCalibur, BD) using specific primary antibodies and Alexa Fluor 647-conjugated secondary antibody (Invitrogen).

### Immunoprecipitation

Immunoprecipitation was performed following previously described methods^70^. NKL cells (4.5 × 10^7^) were surface-biotinylated with EZ-Link™ Sulfo-NHS-Biotin (1 mg/mL, Thermo Fisher Scientific) for 1 h at 4 °C. Cells were lysed in RIPA buffer supplemented with protease inhibitors (Complete™, Roche). Lysate aliquots were immunoprecipitated with 5 µg each of MOPC-21, anti-ITGA4, or ANAP antibodies, followed by capture with Protein G Sepharose (Cytiva). Immune complexes were analyzed by 7% SDS-PAGE and transferred to PVDF membranes. Biotinylated proteins were detected using HRP-streptavidin and Immobilon Forte substrate. For reprobing, membranes were stripped in standard stripping buffer at 50 °C.

### Measurement of epitope masking effect

1 × 10^6^ NKL cells were incubated with 10 µg of each antibody in 100 µL of 1% FCS/PBS/0.05% NaN_3_ on ice for 30 min, and then incubated for another 30 min on ice with 0.1-µg of a labeled (either Alexa Fluor 488 or FITC)-antibody. After washing with cold PBS, samples were subjected to flow cytometry. To confirm that mAb ANAP recognized ITGA4, human peripheral blood was stained with mAb ANAP, anti-CD49d, and anti-CD29 antibodies.

### Scanning electron microscopy

Samples of NKL cells were incubated with mAb ANAP (37 °C; 20 min), and then washed and resuspended in PBS containing 2% FCS. The cell suspension was fixed by adding 0.1 volume of 1% glutaraldehyde in 0.1 M cacodylate buffer (pH 7.3) and incubating the samples at 4 °C for 2 h. The fixed cells were washed with 0.1 M cacodylate buffer, post-fixed in 1% OsO4 (1 h; 4 °C), dehydrated in approximately 50%–100% ethanol, substituted with t-butyl alcohol, dried at − 10 °C under a vacuum, and observed with a high-resolution scanning electron microscope (S-900; Hitachi LTD, Tokyo).

### Statistical analysis

All assays of cytolytic activity were conducted in triplicate, and the data were expressed as mean ± SD. The resulting mean values were < 10% SD. Statistical analyses were performed using SPSS 14.0 software (IBM, NY). The data sets were compared by Student’s t-tests, and *P* values lower than 0.05 were considered significant.

## Supplementary Information

Below is the link to the electronic supplementary material.

Supplementary Material 1

Supplementary Material 2

Supplementary Material 3

Supplementary Material 4

Supplementary Material 5

Supplementary Material 6

Supplementary Material 7

## Data Availability

The datasets used and/or analysed during the current study available from the corresponding author on reasonable request.
