# Supplementary figures and images for: Novel anti-ITGA4 monoclonal antibody induces cell death via large pore formation in NK/T-cell lymphoma cells
Source: Sci Rep. 2025 Dec 30;15:45655. doi: 10.1038/s41598-025-32892-0 (PMC12753658; doi:10.1038/s41598-025-32892-0)

## Supplementary Fig 1

a

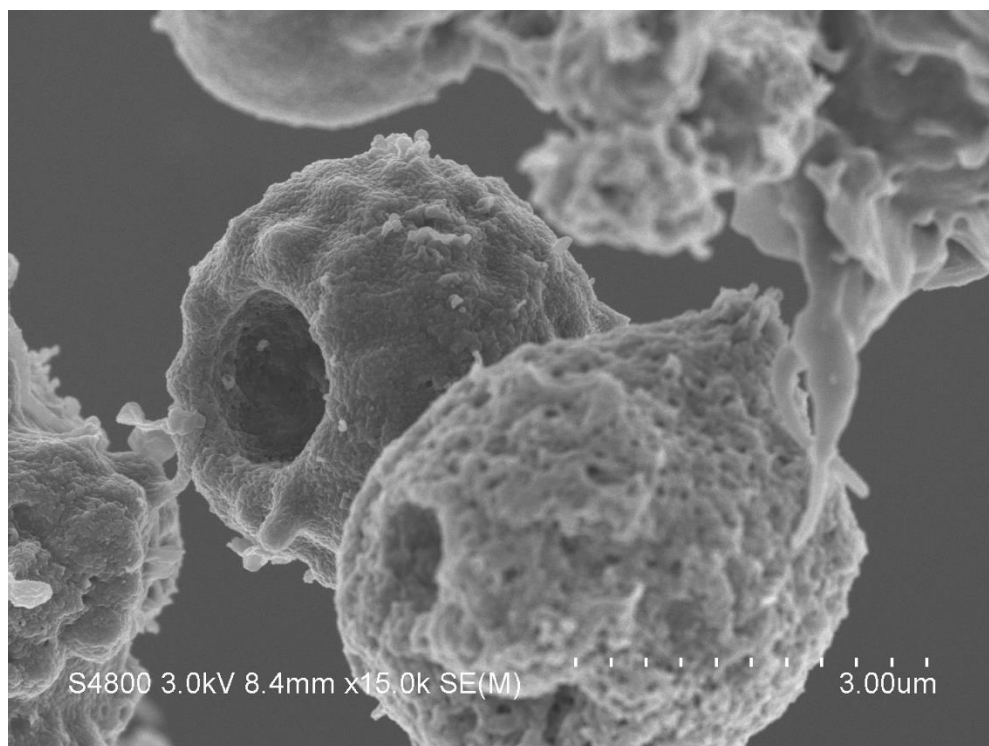

Mouse whole spleen cells

b

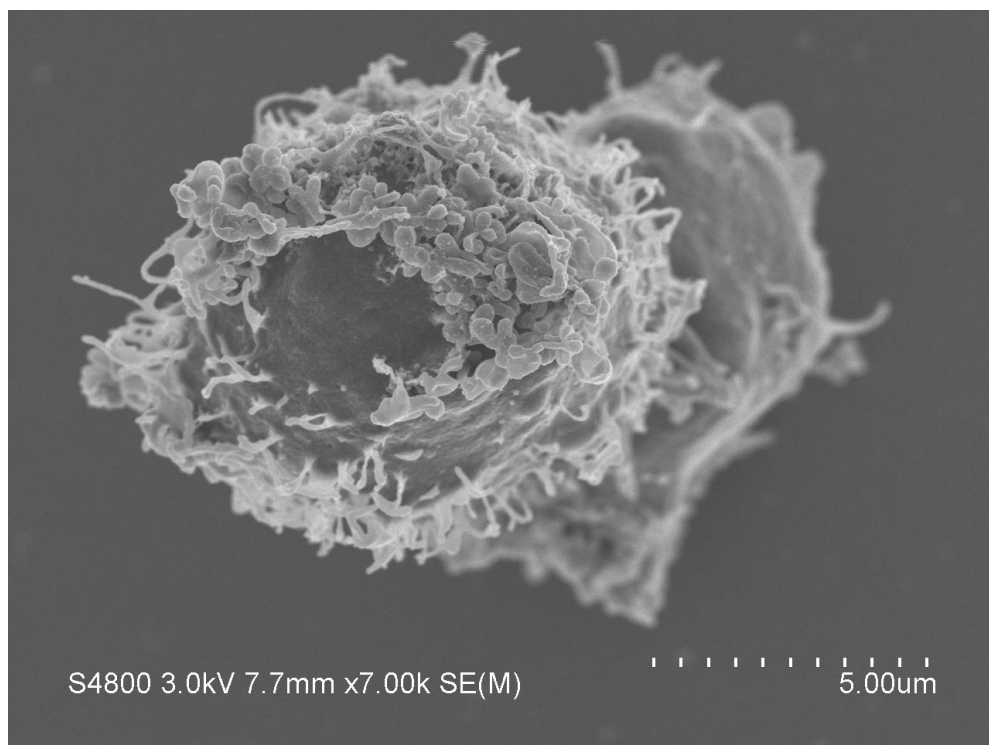

Burkitt lymphoma Raji cells

Supplement: Supplementary file 1 — Supplementary Material 1 [file 41598_2025_32892_MOESM1_ESM.pdf]

# Supplementary Fig 2

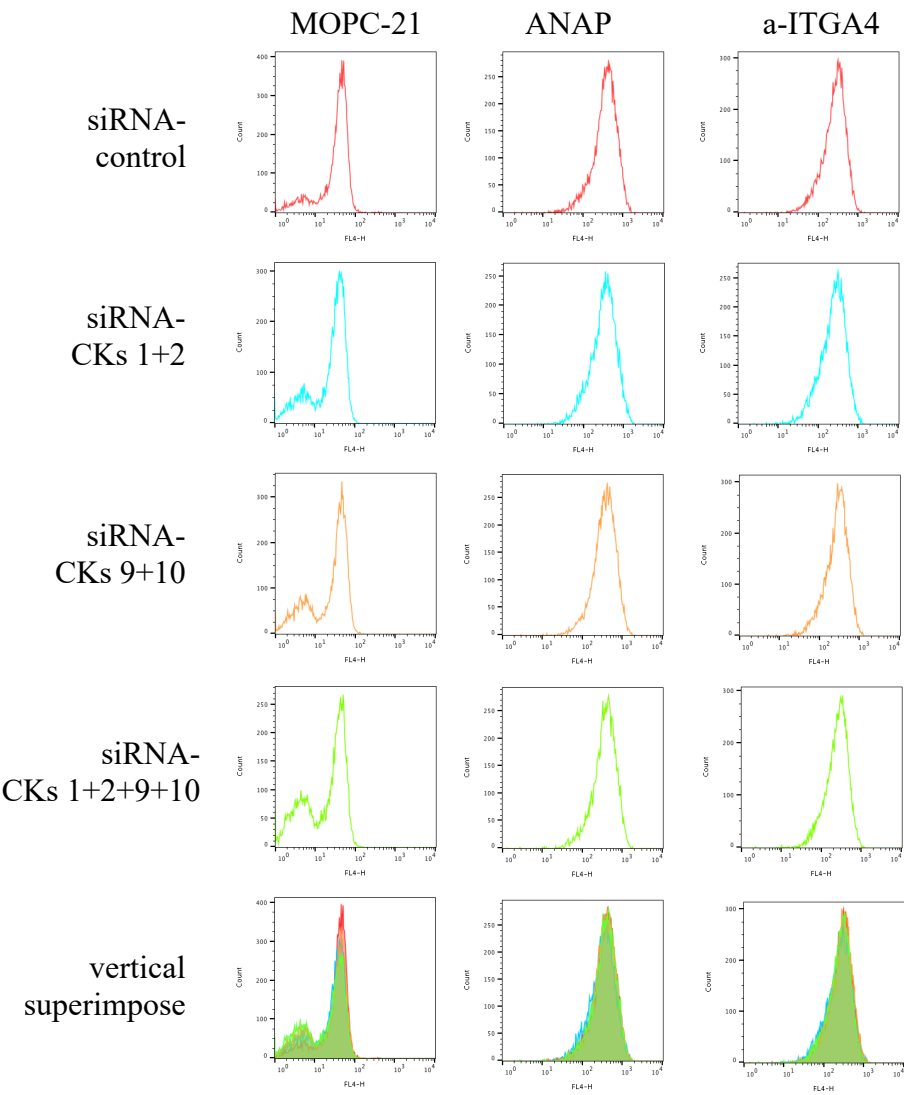

Supplement: Supplementary file 2 — Supplementary Material 2 [file 41598_2025_32892_MOESM2_ESM.pdf]

# Supplementary Fig 3

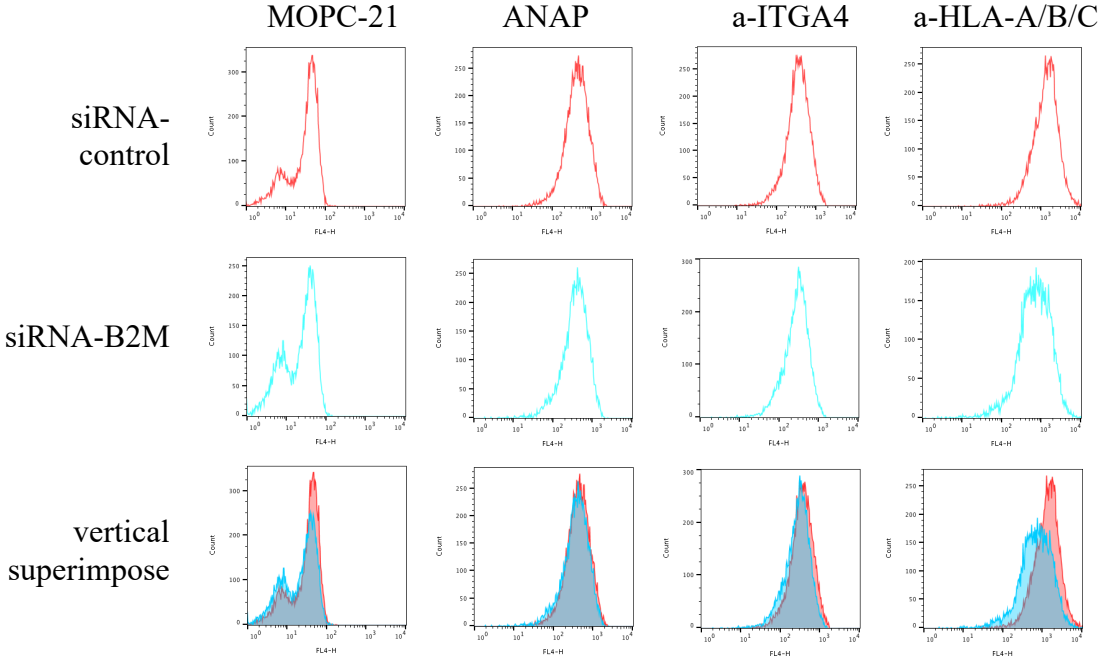

Supplement: Supplementary file 3 — Supplementary Material 3 [file 41598_2025_32892_MOESM3_ESM.pdf]

# Supplementary Fig 4

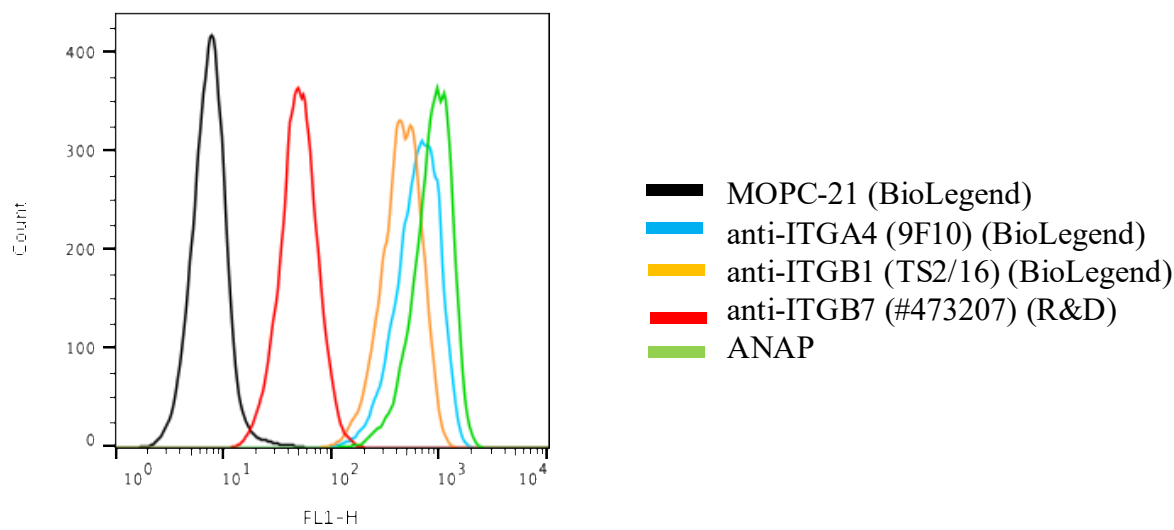

Supplement: Supplementary file 4 — Supplementary Material 4 [file 41598_2025_32892_MOESM4_ESM.pdf]

Supplementary Fig 6

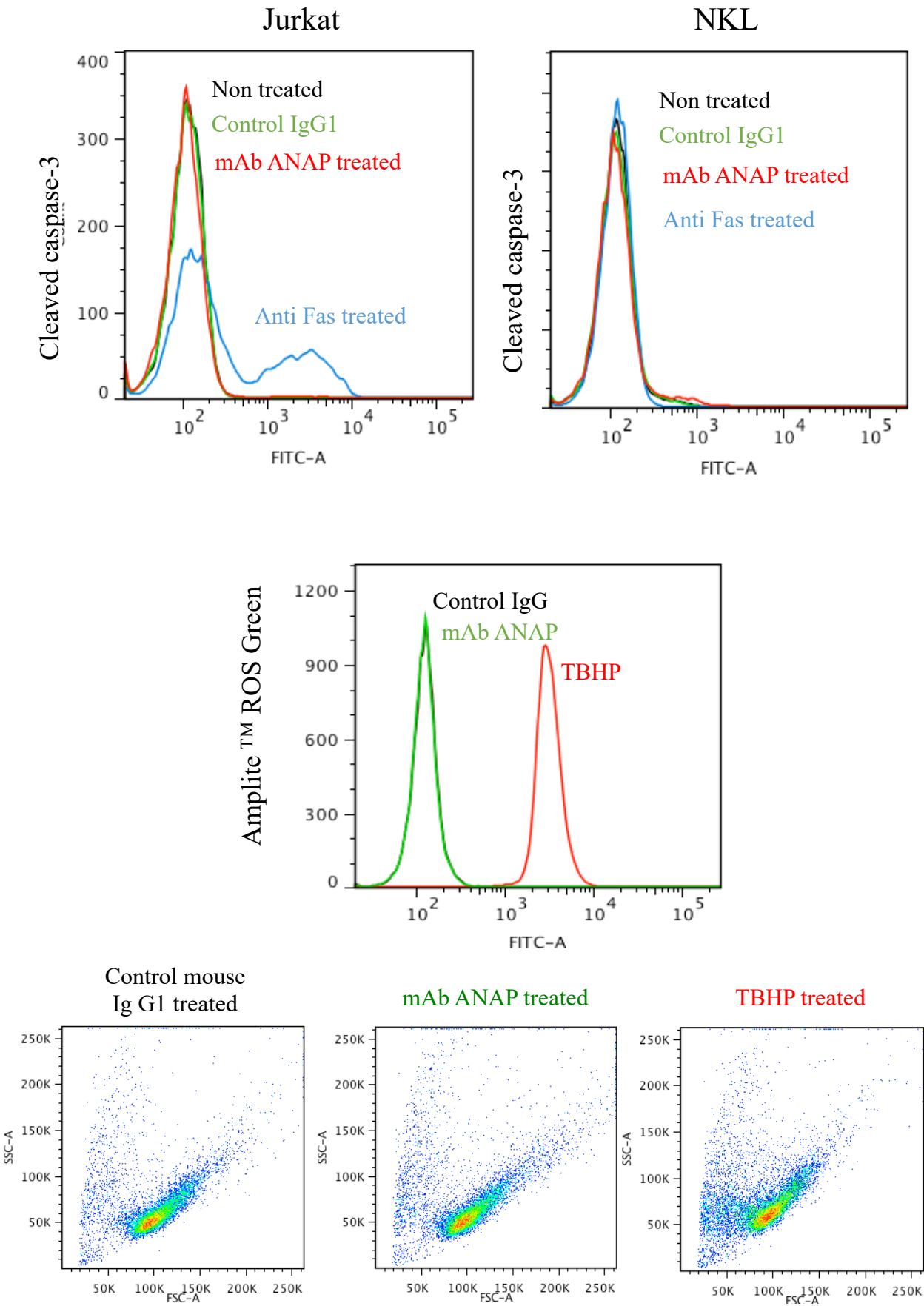

Supplement: Supplementary file 6 — Supplementary Material 6 [file 41598_2025_32892_MOESM6_ESM.pdf]

## Supplementary Fig 7

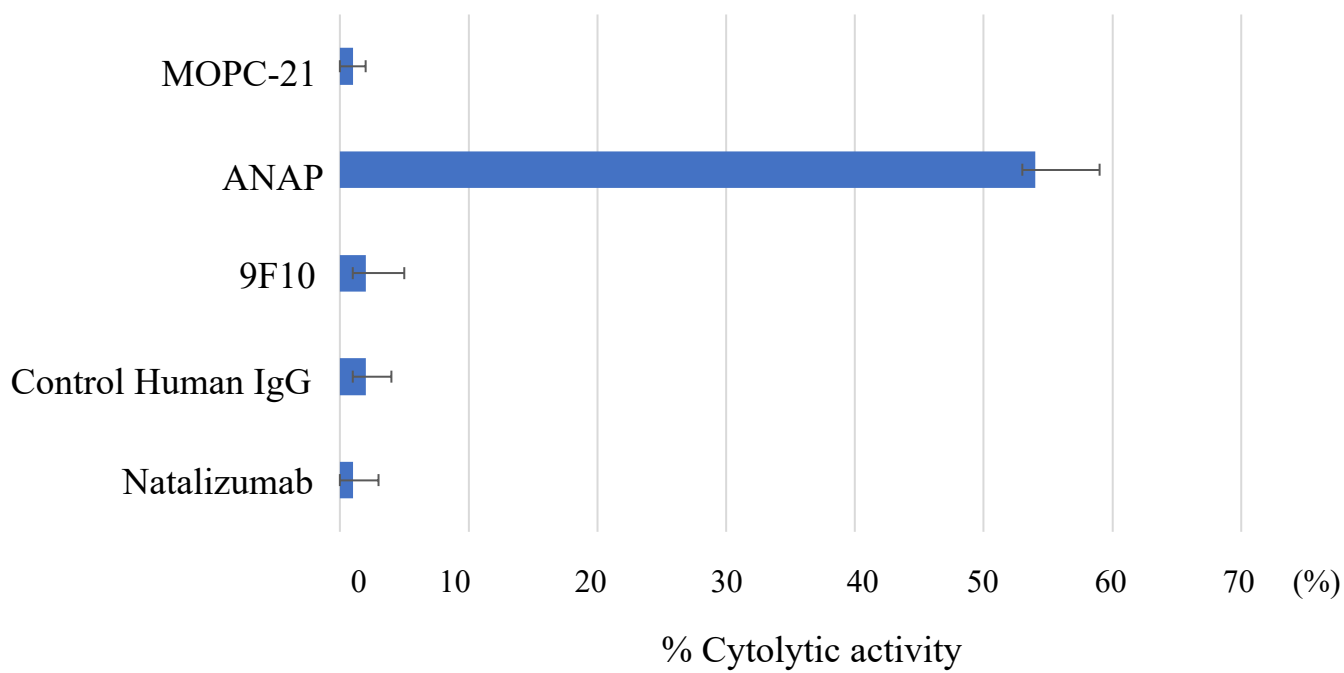

Supplement: Supplementary file 7 — Supplementary Material 7 [file 41598_2025_32892_MOESM7_ESM.pdf]
