# Supplementary material for: Novel anti-ITGA4 monoclonal antibody induces cell death via large pore formation in NK/T-cell lymphoma cells
Source: Sci Rep. 2025 Dec 30;15:45655. doi: 10.1038/s41598-025-32892-0 (PMC12753658; doi:10.1038/s41598-025-32892-0)
Supplement: Supplementary file 5 — Supplementary Material 5 [file 41598_2025_32892_MOESM5_ESM.pdf]

Supplementary Fig 5

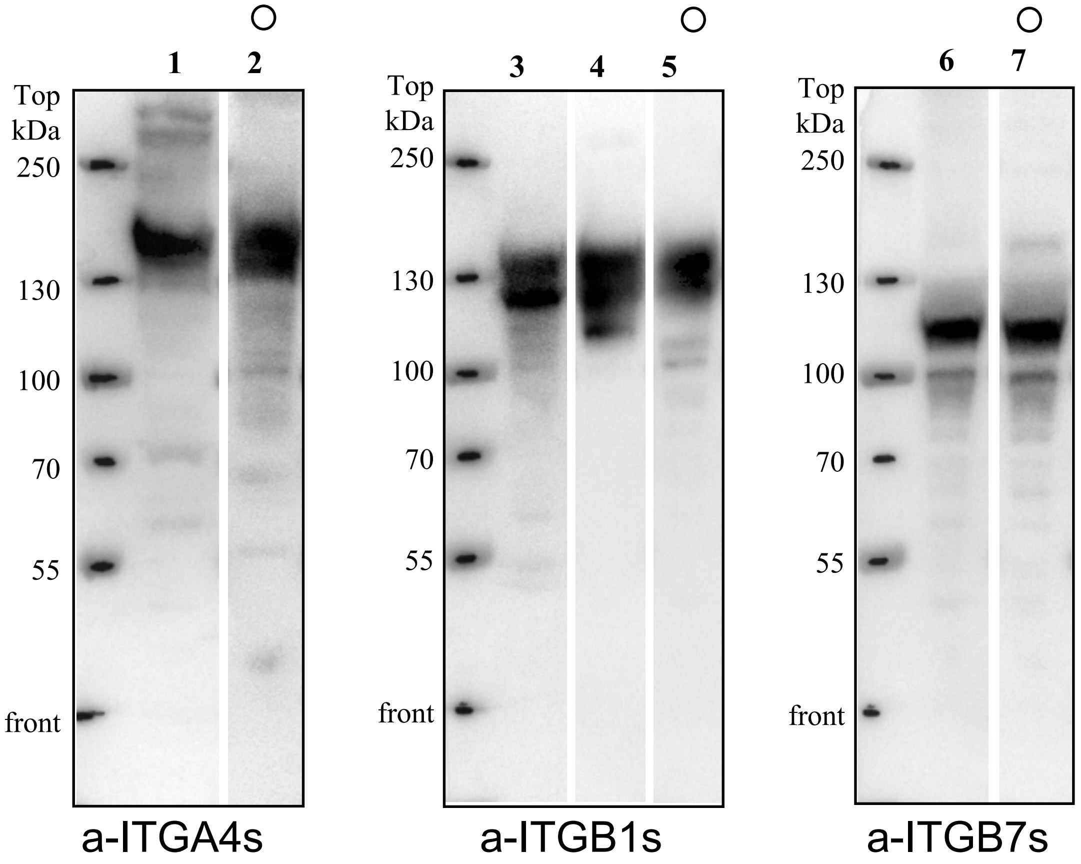

1. Integrin a4 Rabbit polyclonal Ab (Proteintech)
2. Rabbit monoclonal a-Integrin a4/CD49D Ab (EPR1355Y) (Abcam)
3. Integrin b1 Rabbit polyclonal Ab (Proteintech)
4. Integrin b1 Rabbit mAb (D2E5) (Cell Sig)
5. Rabbit monoclonal a-Integrin b1 Ab (EPR1040Y) (Abcam)
6. Integrin b7 Rabbit polyclonal Ab (Proteintech)
7. monoclonal a-Integrin b7 Ab (EP5948) (Abcam)
